# DARS2 Promotes Tumorigenesis and Metastasis by Activating PI3K/Akt/GSK‐3β/β‐Catenin Signaling Pathway in Breast Cancer

**DOI:** 10.1002/kjm2.70267

**Published:** 2026-08-07

**Authors:** Na Wang, Ming Huang

**Affiliations:** ^1^ School of Stomatology and Ophthalmology Xianning Medical College, Hubei University of Science and Technology Xianning Hubei China; ^2^ Department of Thyroid and Breast Surgery Xianning Central Hospital, The First Affiliated Hospital of Hubei University of Science and Technology, The Clinical Research Center for Acute Myocardial Infarction of Hubei Province Xianning Hubei China

**Keywords:** breast cancer, DARS2, epithelial‐mesenchymal transition, PI3K/Akt/GSK‐3β/β‐catenin pathway, tumorigenesis

## Abstract

Aspartyl‐tRNA synthetase 2 (DARS2), the mitochondrial enzyme responsible for aminoacylation of aspartyl‐tRNA, is traditionally known for its effect on protein synthesis. Recently, DARS2 is reported to be implicated in tumorigenesis. However, its specific effect on breast cancer (BC) is poorly understood. The present work employed bioinformatics tools for analyzing DARS2 expression among BC patients and its association with clinicopathological characteristics and patient prognosis. We evaluated DARS2 expression within BC cells and tissues and assessed how DARS2 knockdown or PI3K pathway activation affected cell growth, migration, invasion, apoptosis, as well as cell cycle progression. Mechanistically, we analyzed how DARS2 regulated the PI3K/Akt/GSK‐3β/β‐catenin pathway. Additionally, a xenograft tumor model was constructed to validate our in vitro findings. According to our observations, DARS2 expression significantly increased in BC cells and tissues. Knockdown of DARS2 markedly suppressed cell growth, invasion, migration, and epithelial‐mesenchymal transition (EMT), but enhanced their apoptosis. Mechanistic studies revealed that DARS2 knockdown suppressed the PI3K/Akt/GSK‐3β/β‐catenin pathway, whereas PI3K pathway activation with 740Y‐P reversed the impacts of DARS2 knockdown. In vivo experimental results further verified that DARS2 suppression significantly suppressed tumor growth and metastasis through downregulating the PI3K/Akt/GSK‐3β/β‐catenin pathway. Collectively, these results indicate that DARS2 promotes the growth, migration, invasion, and EMT of BC cells by regulating the PI3K/Akt/GSK‐3β/β‐catenin signaling pathway.

## 1 Introduction

Breast cancer (BC) has become a gynecological neoplasm with the highest malignancy grade worldwide, and its incidence shows a steady and alarming increase over the past several decades [1, 2]. Despite significant advancements in BC treatments, including surgical excision [3], adjuvant chemotherapy [4], radiotherapy [5], immunotherapy [6], and hormone therapy [7], which have collectively improved the five‐year relative survival rates, the prognosis of advanced‐stage BC patients remains discouraging [8, 9]. This persistent challenge is largely associated with the incomplete knowledge regarding the complex molecular mechanisms that underpin BC initiation and progression. A critical gap in our knowledge lies in the identification of key genes and their intricate regulatory networks that drive the aggressive and metastatic behavior of BC cells [10, 11, 12]. Therefore, it is of paramount importance to discover and thoroughly characterize novel genes that are aberrantly expressed in BC and contribute to its malignant progression. These insights are essential for developing innovative targeted treatments to efficiently combat the devastating disease.

Aminoacyl‐tRNA synthetases (ARSs) exert an essential effect on facilitating protein synthesis through binding amino acids to corresponding transfer RNA (tRNA) molecules [13]. Recent research has revealed that ARSs, in addition to the critical effect on protein synthesis, are related to various biological events, such as angiogenesis, cellular homeostasis, and tumorigenesis regulation [14, 15]. Among the ARSs family, aspartyl‐tRNA synthetase 2 (DARS2) stands out as a particularly intriguing enzyme. DARS2, as the mitochondrial enzyme, primarily functions in aminoacylation of aspartic acid‐tRNA, a critical step in protein synthesis within the mitochondria [16, 17]. However, emerging evidence has suggested that DARS2's role extends beyond its enzymatic activity. It appears to contribute to other cellular functions, especially in the context of tumor development and progression. DARS2 is recently suggested to be involved in several cancer types, including hepatocellular carcinoma (HCC) [18], lung adenocarcinoma (LUAD) [19], bladder cancer [20], and esophageal cancer [21]. In these cancers, aberrant expression of DARS2 is closely linked to malignant transformation and tumor progression. According to the above studies, DARS2 exerts an essential effect on oncogenesis, potentially serving as a novel anti‐tumor therapeutic target. However, despite these preliminary reports, the precise relationship between DARS2 and the malignant phenotype of BC remains largely unexplored. Typically, the lack of comprehensive studies specifically addressing the role of DARS2 in BC represents a significant gap in the knowledge regarding the BC‐related molecular mechanisms.

Therefore, the current work aimed to thoroughly investigate DARS2's effect on BC growth, migration, and invasion. Tumor cells were used in combination with animal models for elucidating its functional impact on BC progression. Furthermore, we sought to unravel those molecular mechanisms of DARS2 in facilitating BC pathogenesis. Our findings may help uncover a novel anti‐BC therapeutic target, shedding novel lights on developing more effective and targeted treatment strategies.

## 2 Materials and Methods

### 2.1 Bioinformatics Analysis

First, by utilizing the GEPIA2 online database (http://gepia2.cancer‐pku.cn/), differential DARS2 expression in breast invasive carcinoma (BRCA) versus non‐carcinoma tissues was assessed with The Cancer Genome Atlas (TCGA) and Genotype‐Tissue Expression (GTEx) datasets. Furthermore, we employed the UALCAN web portal (http://ualcan.path.uab.edu) for assessing DARS2 expression based on various clinicopathological parameters, including sample type, individual cancer stages, cancer subclasses, and nodal metastasis status.

### 2.2 Patients and Tissue Samples

Between February 2020 and February 2022, 50 tumor specimens and matched non‐carcinoma tissues were obtained in BC cases undergoing mastectomy at Xianning Central Hospital, The First Affiliated Hospital of Hubei University of Science and Technology. This study gained approval from the Ethics Committees of the hospital and was implemented in line with the Declaration of Helsinki. Informed consent was obtained from each patient.

### 2.3 Cell Lines and Culture

Human BC cells, including T47D, MCF‐7, and MDA‐MB‐231, along with the healthy breast epithelial cells MCF10A, were provided by the Cell Bank of the Chinese Academy of Sciences (Shanghai, China) and maintained within Dulbecco's Modified Eagle Medium (DMEM, Gibco, Rockville, MD, USA) that contained 10% fetal bovine serum (FBS) as well as 1% antibiotic/antimycotic solution under 37°C and 5% CO_2_ conditions.

### 2.4 Quantitative Real‐Time Polymerase Chain Reaction (qRT‐PCR)

TRIzol reagent (Invitrogen, Carlsbad, CA, USA) was employed for extracting total tissue and cellular RNA. Thereafter, cDNA was prepared with PrimeScript RT Reagent Kit (Takara, Shiga, Japan). By using SYBR Premix Ex Taq II (Takara), qPCR was performed on the ABI PRISM 7500 Sequence Detection System (Applied Biosystems, Carlsbad, CA, USA). All primers used for qRT‐PCR were as follows: for DARS2: 5′‐GGAGAGTTGCGTTCGTCTCA‐3′ (forward), 5′‐GATTCCACAGGG GCTTCACA‐3′ (reverse); for GAPDH: 5′‐AATGGGCAGCCGTTAGGAAA‐3′ (forward), 5′‐GCGCCCAATACGACCAAATC‐3′ (reverse). GAPDH was the endogenous reference. We adopted the 2^−ΔΔCt^ approach for calculating RNA expression.

### 2.5 Transfection and Treatment

When cells were grown to 80%–90% confluence, cell passage was conducted. Transfection experiments were carried out during the logarithmic growth phase. In brief, si‐DARS2 (sense: 5′‐AGACAGACAGCCUGAGUUUTT‐3′; antisense: 5′‐AAACUCAGGCUGUCUGUCUTT‐3′) or negative control siRNA (si‐NC) (sense: 5′‐UUCUCCGAACGUGUCACGUTT‐3′; antisense: 5′‐ACGUGACACGUUCGGAGAATT‐3′) was transfected in MCF‐7 and MDA‐MB‐231 cells. In some experiments, we dissolved the PI3K agonist 740Y‐P (Catalog #: 1983; R&D Systems, Minneapolis, MN, USA) in DMSO for preparing a 10 mM stock solution, and added serum‐free medium for dilution to a 50 μg/mL working concentration to treat transfected cells for 90 min as previously described [22, 23].

### 2.6 Cell Counting Kit‐8 (CCK‐8) Assay

By utilizing the CCK‐8 assay kit (Beyotime Biotechnology, Shanghai, China), we assessed cell viability. To be specific, transfected cells were harvested and planted into 96‐well plates at 2 × 10^3 cells/mL. At 24, 48, and 72 h post‐incubation under 37°C and 5% CO_2_ conditions, CCK‐8 solution (10 μL) was poured into every well for another 2 h of incubation. Absorbance was measured with the microplate reader (Bio‐Rad, Hercules, CA, USA) at 450 nm.

### 2.7 Colony Formation Assay

To evaluate cell proliferation, a colony formation assay was conducted. In brief, 2000 resuspended cells were plated into 6‐well plates and cultured for a 14‐day period. Subsequently, they were fixed with 4% paraformaldehyde for 15 min and stained with 0.1% crystal violet for 30 min. Later, the stained colonies were washed and photographs were taken.

### 2.8 Flow Cytometry for Cell Cycle and Apoptosis Analyses

Cell apoptosis was assessed with an Annexin V‐FITC apoptosis detection kit (Beyotime Biotechnology). Cells were rinsed after 48 h of starvation, followed by resuspension within staining buffer at 1 × 10^6 cells/mL. Then, we utilized a FACSCanto flow cytometer (BD Biosciences, San Diego, CA, USA) for analysis. To this end, following fixation using 70% ethanol under 4°C overnight, cells were rinsed prior to resuspension within PI/RNase buffer (500 μL), 15 min of incubation under ambient temperature, and flow cytometric analysis.

### 2.9 Wound Healing Assay

To assess cell motility, we conducted a scratch assay. In brief, both transfected and control cells were grown in 6‐well plates that contained 1% FBS‐containing medium. After reaching 100% confluence, we employed a 10 μL pipette tip for creating a scratch. Following PBS washing, cells were imaged at various time intervals. The width of the wound was measured and compared to baseline values to determine the relative migration rate.

### 2.10 Transwell Assays

Transwell chambers (Costar‐Corning, New York, USA), with/without Matrigel coating, were adopted for analyzing cell migration and invasion. Briefly, cells (5 × 10^4^) were inoculated into the top chamber containing serum‐free medium, whereas 10% FBS‐containing medium was introduced into the bottom chamber. Following the removal of non‐migrating or non‐invading cells at 24 h post‐incubation, those invading cells were processed through fixation, crystal violet staining, and counting with a microscope (Olympus Corporation, Tokyo, Japan).

### 2.11 Western Blotting

Immunoblotting was implemented following specific procedures. In brief, RIPA reagent (Beyotime Biotechnology) was utilized for total protein isolation. Protein aliquots (30 μg) were subjected to 12% SDS‐PAGE for separation before transfer on PVDF membranes (Millipore, Bedford, MA, USA). Following a 1‐h blocking period using 5% nonfat milk at room temperature, these membranes were incubated with primary antibodies (1:1000 dilution) overnight at 4°C. After washing, membranes were further incubated with secondary antibody (1:2000 dilution, #7074, Cell Signaling Technology) for 1 h at room temperature. Protein bands were detected with the ECL detection system (Beyotime Biotechnology) and examined with ImageJ software (NIH, Bethesda, MD, USA). Notably, all primary antibodies utilized in the current work were anti‐DARS2 (ab154606, Abcam, Cambridge, UK); anti‐Cyclin D1 (#55506), anti‐CDK2 (#2546), anti‐CDK4 (#12790), anti‐CDK6 (#30483), anti‐E‐cadherin (#3195), anti‐N‐cadherin (#4061), anti‐Vimentin (#5741), anti‐Bcl‐2 (#3498), anti‐Bax (#2772), cleaved caspase‐3 (#9664), anti‐p‐PI3K (#17366), anti‐PI3K (#4292), anti‐p‐AKT (#4060), anti‐AKT (#9272), anti‐GSK‐3β (#9315), anti‐β‐catenin (#8480), and anti‐GAPDH (#2118) (all from Cell Signaling Technology).

### 2.12 In Vivo Tumorigenesis and Metastasis Assays

We acquired the 5‐week‐old female BALB/c nude mice in Shanghai Experimental Animal Center and raised them under sterile conditions. The animal procedures gained approval from the Institutional Animal Care and Use Committee of Xianning Central Hospital, The First Affiliated Hospital of Hubei University of Science and Technology.

For tumorigenesis assay, MDA‐MB‐231 cells (3 × 10^6^) following stable si‐DARS2 or control plasmid transfection were given subcutaneously in flanks of mice. Tumor diameters were measured at 7‐day intervals to monitor tumor growth. Tumor volumes could be computed as follows: volume = 0.5 × length × width^2^. At 28 days later, tumor tissues were excised following euthanasia, and the weights were measured.

In metastasis assay, MDA‐MB‐231 cells (8 × 10^4^) receiving stable si‐DARS2 or si‐NC transfection were given in lateral tail veins of nude mice. At 6 weeks later, lungs were collected to assess the number of metastatic nodules.

### 2.13 Immunohistochemistry (IHC)

Tissues collected from BC patients and mouse flanks were immersed within 4% paraformaldehyde, followed by paraffin embedding and sectioning in 5‐μm slices. Thereafter, the slices were deparaffinized, rehydrated, and treated for inhibiting endogenous peroxidase activity and non‐specific binding. Afterwards, slices were first incubated with rabbit polyclonal anti‐DARS2 (1:250 dilution, ab154606, Abcam) and anti‐Ki‐67 (1:500 dilution, ab15580, Abcam) primary antibodies overnight, then probed using HRP‐labeled goat anti‐rabbit IgG H&L secondary antibody (1:300 dilution, ab7090, Abcam), and finally stained with the DAB substrate kit (ab64238, Abcam). Staining results were analyzed under a microscope.

### 2.14 Statistical Analyses

Each assay was conducted thrice at least. Data were represented by means ± standard deviation (SD). Between‐group comparison was examined with Student's *t*‐test, while among‐group one with ANOVA followed by a post hoc test. We employed GraphPad Prism 8 (GraphPad Software, San Diego, CA, USA) to complete statistical analysis. *p* < 0.05 (two‐tailed) suggested significant differences.

## 3 Results

### 3.1 DARS2 Expression Increases Within BC and Is Related to Unfavorable Prognostic Outcome

**FIGURE 1:**
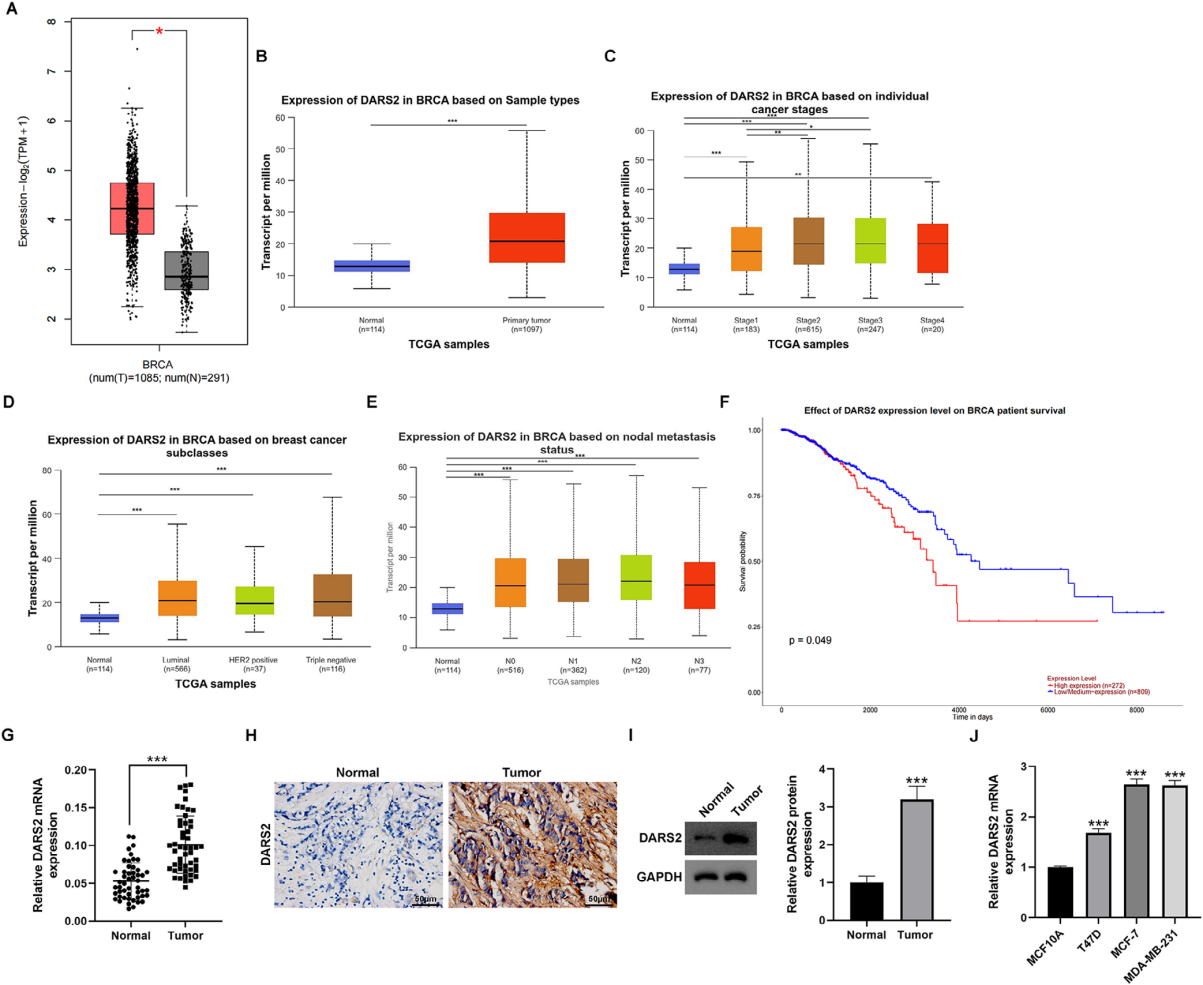
DARS2 is upregulated in BC and correlates with poor prognosis. (A) Analysis of the TCGA database using GEPIA2 revealed significantly higher DARS2 mRNA levels in BC tissues compared to normal tissues. (B) UALCAN analysis of the TCGA dataset further confirmed the upregulation of DARS2 in BC tissues. (C‐F) UALCAN analysis indicated that elevated DARS2 expression was positively correlated with advanced tumor stages (C), molecular subclasses (D), lymph node metastasis (E), and poorer overall survival in BC patients (F). (G) qRT‐PCR analysis of 50 paired BC and adjacent normal tissues demonstrated significant upregulation of DARS2 mRNA in tumor tissues. (H) IHC analysis showed DARS2 protein expression in BC tissues and normal tissues (Scale bar = 50 μm). (I) Western blotting showed markedly higher DARS2 protein levels in BC tissues compared to normal tissues. (J) DARS2 expression was significantly higher in BC cell lines (T47D, MCF‐7, MDA‐MB‐231) than in the normal breast epithelial cell line MCF10A. Data are presented as mean ± SD. *p < 0.05, **p < 0.01, ***p < 0.001.

To elucidate DARS2's effect on BC, its expression was evaluated using public databases. From TCGA database analysis through GEPIA2 and UALCAN, DARS2 mRNA levels markedly increased within BC compared with matched non‐carcinoma samples (Figure 1A,B). As revealed by further UALCAN analysis, DARS2 expression was positively correlated with tumor stages, molecular subclasses, lymph node metastasis, and overall survival (Figure 1C–F).

To validate these findings, DARS2 expression was examined within BC cells and tissues. qRT‐PCR assay was conducted for analyzing its expression in 50 bc and 50 matched non‐carcinoma tissues, which confirmed significant DARS2 upregulation in tumor tissues (Figure 1G). Furthermore, according to IHC assay, DARS2 expression increased within tumor relative to matched non‐carcinoma tissues (Figure 1H). Likewise, Western blotting revealed markedly elevated DARS2 protein levels within tumor compared with matched non‐carcinoma tissues (Figure 1I). Moreover, DARS2 expression remarkably increased within BC cells (T47D, MCF‐7, MDA‐MB‐231) relative to healthy breast epithelial cells (MCF10A) (Figure 1J). The above results collectively reveal DARS2 as the novel biomarker for BC.

### 3.2 DARS2 Silencing Inhibits BC Cell Proliferation and Induces Cell Cycle Arrest

**FIGURE 2:**
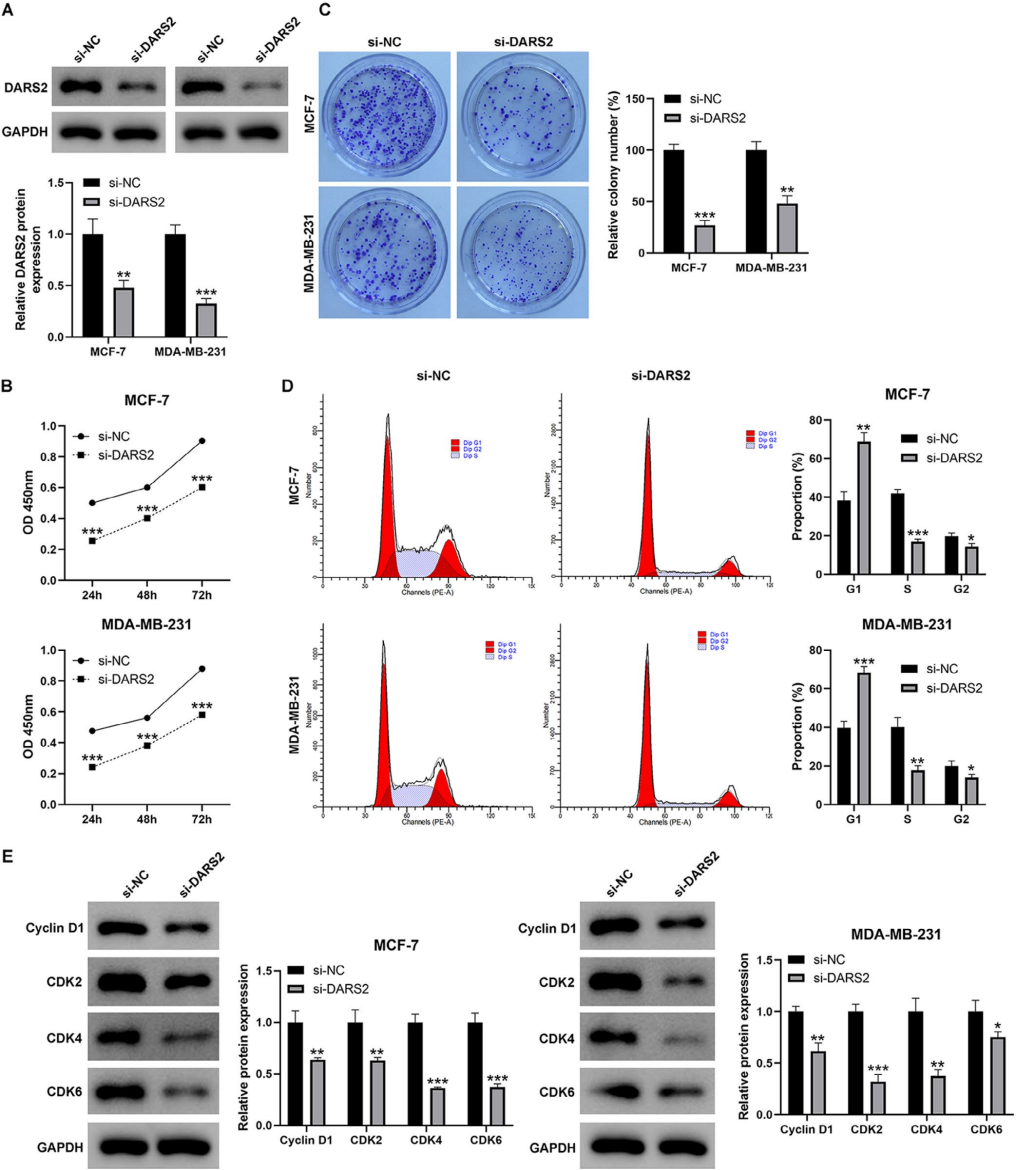
DARS2 knockdown inhibits BC cell proliferation and induces G1/S cell cycle arrest. (A) Western blotting confirmed efficient knockdown of DARS2 in MCF‐7 and MDA‐MB‐231 cells transfected with DARS2 siRNA. (B) CCK‐8 assay showed that DARS2 knockdown significantly inhibited the proliferation of MCF‐7 and MDA‐MB‐231 cells. (C) Colony formation assay demonstrated that DARS2 knockdown reduced the colony‐forming ability of BC cells. (D) Flow cytometry analysis revealed that DARS2 knockdown induced G1/S cell cycle arrest in both MCF‐7 and MDA‐MB‐231 cells. (E) Western blotting showed that DARS2 knockdown decreased the expression of key cell cycle regulators, including Cyclin D1, CDK2, CDK4, and CDK6. Data are presented as mean ± SD. *p < 0.05, **p < 0.01, ***p < 0.001 versus si‐NC group.

To explore DARS2's function within BC cells, loss‐of‐function experiments were carried out using siRNA targeting DARS2. Western blotting confirmed efficient silencing of DARS2 within MCF‐7 and MDA‐MB‐231 cells (Figure 2A). As suggested by CCK‐8 and colony formation assays, DARS2 silencing dramatically inhibited cell growth (Figure 2B,C). Based on flow cytometry results, DARS2 silencing arrested the cell cycle at G1/S phase in these cells (Figure 2D). Consistently, Western blotting showed that DARS2 silencing decreased the expression of key cell cycle regulators, including CDK2, CDK4, CDK6, and Cyclin D1 (Figure 2E). These data demonstrate that DARS2 silencing inhibits BC cell proliferation by inducing G1/S phase arrest.

### 3.3 DARS2 Silencing Suppresses BC Cell Migration, Invasion, and Epithelial‐Mesenchymal Transition (EMT)

**FIGURE 3:**
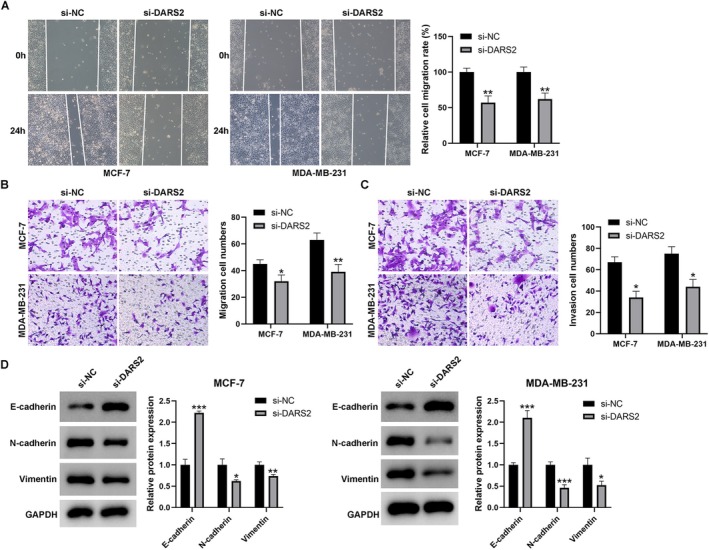
DARS2 knockdown suppresses BC cell migration, invasion, and EMT. (A) Wound healing assay demonstrated that DARS2 knockdown significantly inhibited the migration of MCF‐7 and MDA‐MB‐231 cells. (B) Transwell migration assay confirmed the inhibitory effect of DARS2 knockdown on BC cell migration. (C) Transwell invasion assay showed that DARS2 knockdown significantly reduced the invasion ability of BC cells. (D) Western blotting revealed that DARS2 knockdown increased the expression of the epithelial marker E‐cadherin and decreased the expression of the mesenchymal markers N‐cadherin and vimentin, indicating suppression of EMT. Data are presented as mean ± SD. *p < 0.05, **p < 0.01, ***p < 0.001 versus si‐NC group.

Next, we analyzed how DARS2 silencing affected BC cell migration and invasion. From scratch and Transwell assays, DARS2 silencing markedly suppressed migration and invasion within BC cells (Figure 3A–C). Western blotting revealed that DARS2 silencing upregulated E‐cadherin (an epithelial marker) but decreased N‐cadherin and vimentin expression (mesenchymal markers), indicating suppression of EMT (Figure 3D). Collectively, the above results illustrate the important effect of DARS2 on enhancing BC cell migration, invasion, and EMT.

### 3.4 DARS2 Silencing Promotes BC Cell Apoptosis

**FIGURE 4:**
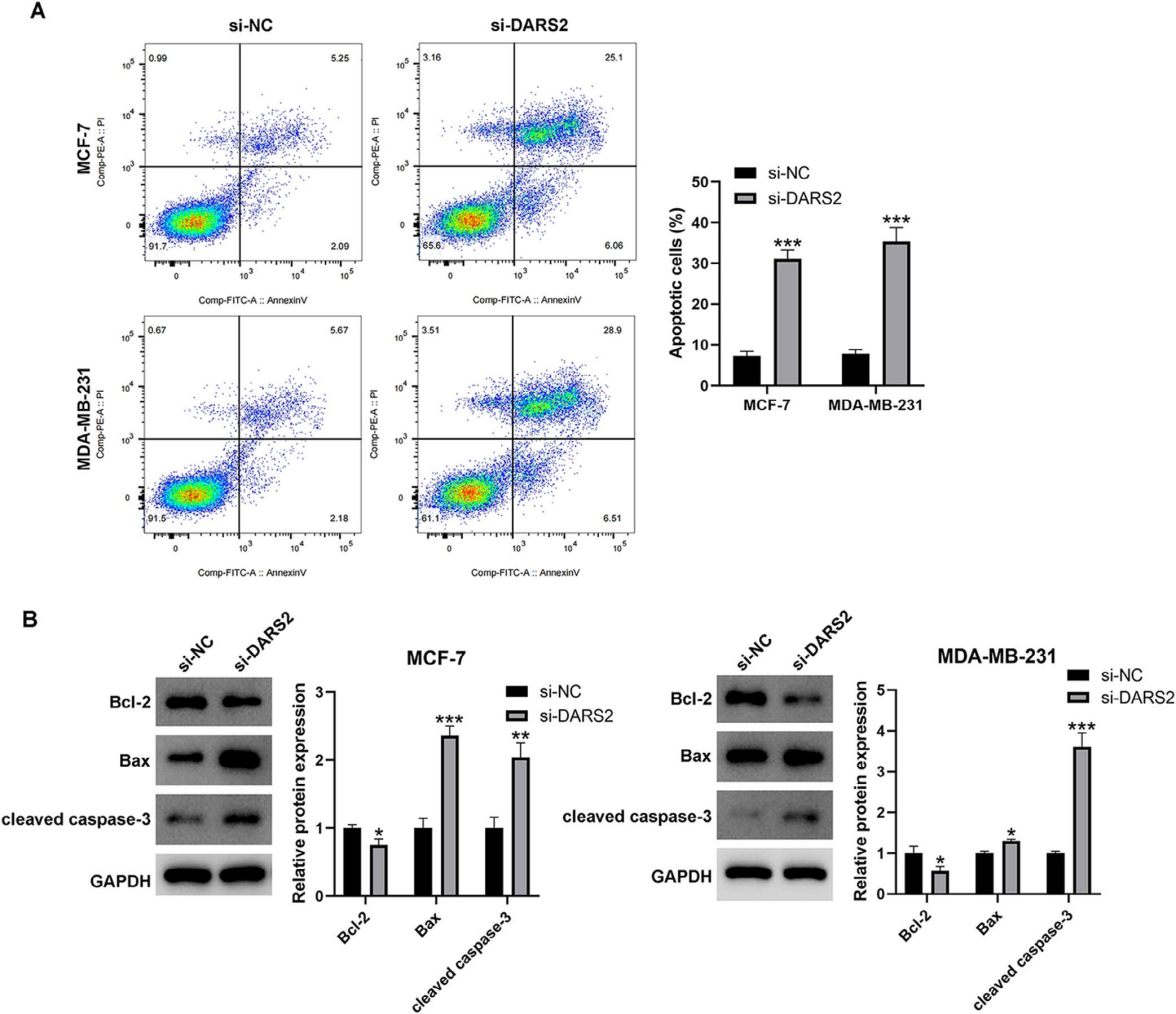
DARS2 knockdown promotes BC cell apoptosis. (A) Flow cytometry analysis showed that DARS2 knockdown significantly increased the apoptosis rate of MCF‐7 and MDA‐MB‐231 cells. (B) Western blotting demonstrated that DARS2 knockdown upregulated the expression of the pro‐apoptotic protein Bax and downregulated the expression of the anti‐apoptotic protein Bcl‐2. Data are presented as mean ± SD. *p < 0.05, **p < 0.01, ***p < 0.001 versus si‐NC group.

To analyze whether DARS2 silencing induced apoptosis in BC cells, we performed flow cytometry analysis. The data revealed that DARS2 silencing apparently promoted apoptosis in BC cells (Figure 4A). From Western blotting, DARS2 silencing elevated Bax (the pro‐apoptotic protein) and decreased Bcl‐2 (the anti‐apoptotic protein) levels (Figure 4B). These results indicate that DARS2 depletion stimulates apoptosis in BC cells.

### 3.5 DARS2 Promotes BC Progression Through the PI3K/Akt/GSK‐3β/β‐Catenin Pathway

**FIGURE 5:**
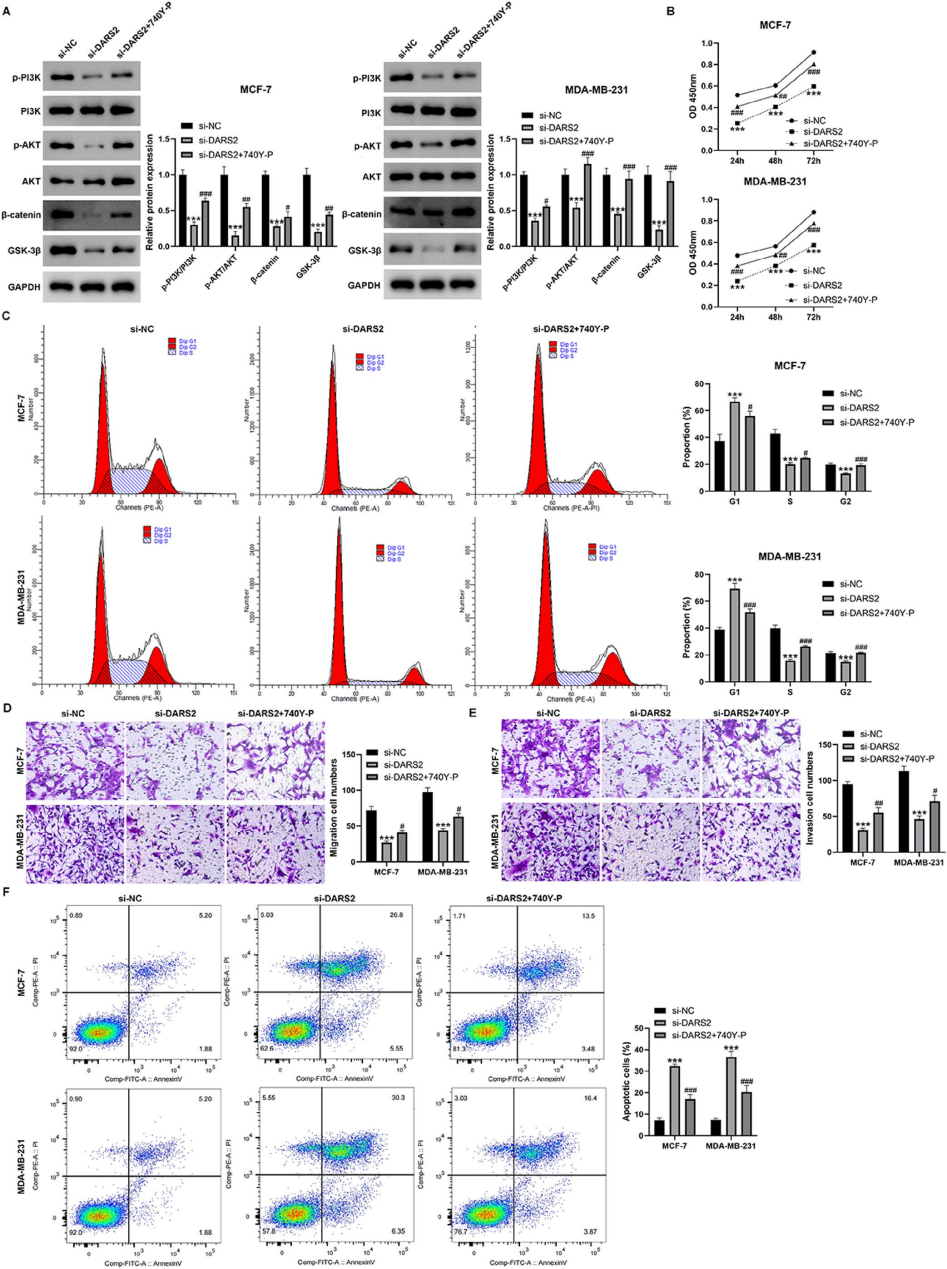
DARS2 promotes BC progression via the PI3K/Akt/GSK‐3β/β‐catenin pathway. (A) Western blotting showed that DARS2 knockdown decreased the phosphorylation of PI3K and Akt and reduced the expression of GSK‐3β and β‐catenin in MCF‐7 and MDA‐MB‐231 cells. The addition of the PI3K agonist 740Y‐P rescued the effects of DARS2 knockdown. (B) CCK‐8 assay demonstrated that 740Y‐P treatment increased cell viability in DARS2‐knockdown cells. (C) Flow cytometry analysis revealed that 740Y‐P reduced G1/S phase arrest induced by DARS2 knockdown. (D, E) Transwell assays showed that 740Y‐P enhanced the migration and invasion of DARS2‐knockdown cells. (F) Flow cytometry analysis showed that 740Y‐P decreased apoptosis in DARS2‐knockdown cells. Data are presented as mean ± SD. ***p < 0.001 versus si‐NC group; ^#^ *p* < 0.05, ^##^ *p* < 0.01, ^###^ *p* < 0.001 versus si‐DARS2 group.

To explore the exact mechanism of DARS2 in promoting BC progression, the PI3K/Akt/GSK‐3β/β‐catenin pathway, which is frequently dysregulated within cancer, was our focus. In BC cells, DARS2 silencing reduced PI3K and Akt phosphorylation as well as GSK‐3β and β‐catenin levels, suggesting an inhibitory effect on this pathway (Figure 5A). Apart from activating this pathway (Figure 5A), adding 740Y‐P effectively rescued the effects of DARS2 knockdown, leading to increased cell viability (Figure 5B), reduced G1/S phase arrest (Figure 5C), enhanced cell migration and invasion (Figure 5D,E), and decreased apoptosis (Figure 5F). These findings further support the notion that DARS2 exerts its oncogenic effects in BC cells through the PI3K/Akt/GSK‐3β/β‐catenin pathway.

### 3.6 DARS2 Silencing Inhibits Tumor Proliferation and Metastasis In Vivo

**FIGURE 6:**
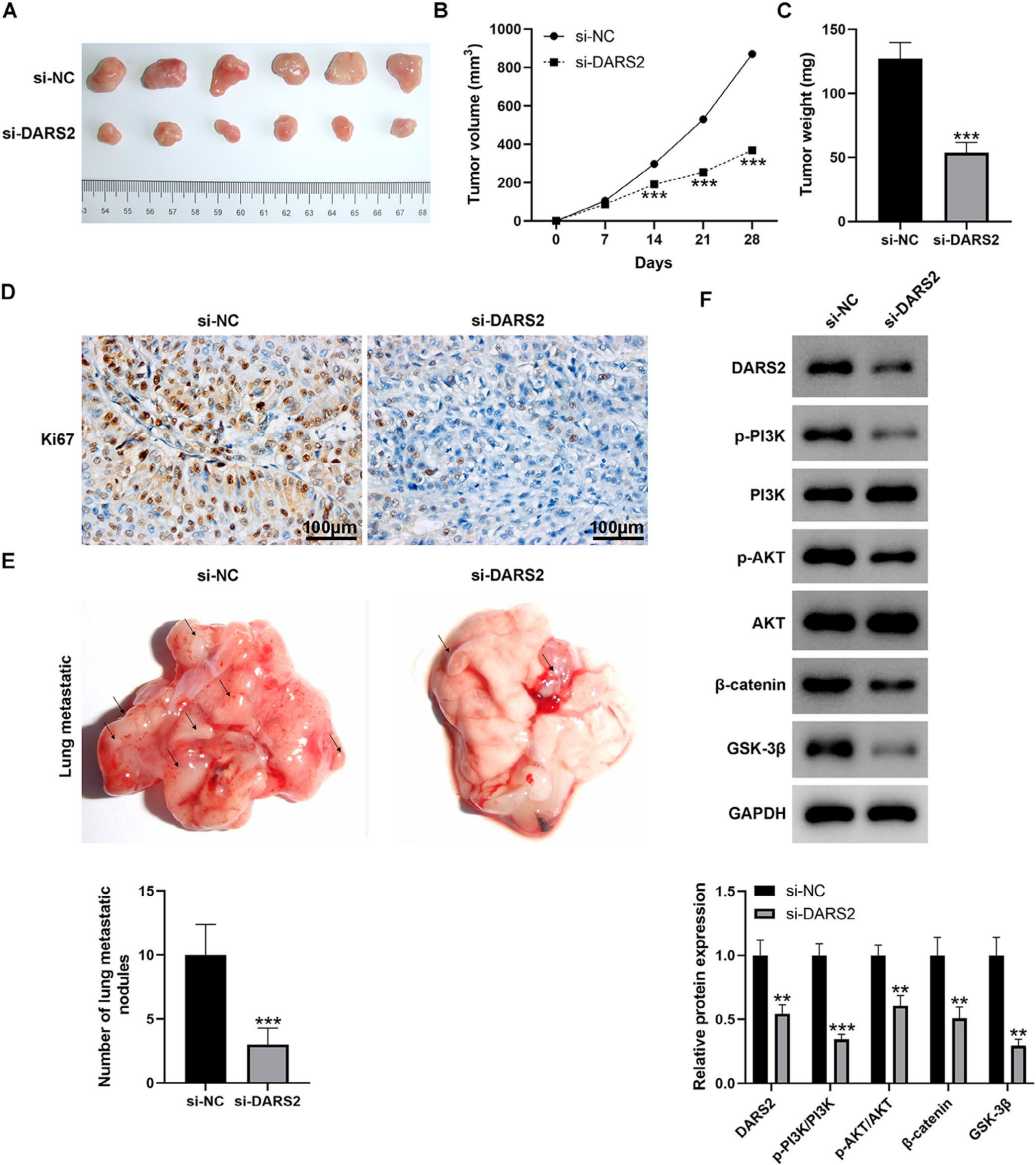
DARS2 knockdown inhibits tumor growth and metastasis in vivo. (A–C) DARS2 knockdown significantly inhibited tumor growth in a xenograft model, as evidenced by reduced tumor volume and weight. (D) Immunohistochemical staining showed decreased expression of the proliferation marker Ki‐67 in tumors with DARS2 knockdown. (E) Western blotting confirmed the downregulation of DARS2 and key proteins in the PI3K/Akt/GSK‐3β/β‐catenin pathway in tumors with DARS2 knockdown. (F) DARS2 knockdown significantly reduced the number of lung metastatic nodules in a lung metastasis model. Data are presented as mean ± SD. **p < 0.01, ***p < 0.001 versus si‐NC group.

To further verify the above results, we constructed a xenograft tumor model through subcutaneous injection of DARS2‐silenced MDA‐MB‐231 cells in mice. The decreased tumor weight and volume suggested that DARS2 silencing remarkably suppressed tumor proliferation (Figure 6A–C). IHC analysis revealed downregulation of Ki‐67, a proliferation marker, within tumors with DARS2 knockdown (Figure 6D). Western blotting confirmed the downregulation of DARS2 and key proteins related to the PI3K/Akt/GSK‐3β/β‐catenin pathway inside tumors with DARS2 knockdown (Figure 6E). Besides, we constructed a lung metastasis model by injecting DARS2‐silenced MDA‐MB‐231 cells in the tail vein of mice. DARS2 knockdown significantly decreased the lung metastatic nodule quantity (Figure 6F).

Taken together, DARS2 expression increases within BC, which exerts a key effect on enhancing tumor progression by the PI3K/Akt/GSK‐3β/β‐catenin pathway. This work sheds new light on those molecular mechanisms related to BC progression and highlights DARS2 as the candidate therapeutic target for this devastating disease.

## 4 Discussion

The significant improvement in primary BC patient survival is attributed to enhanced mammography screening and surgical advancements. However, it remains challenging to manage metastatic BC [24]. Treatment options are severely limited in the context of distant metastases of BC (including lung, brain, and liver metastases). This may be due to the complex and multifaceted nature of the metastatic process, which involves a series of intricate molecular events [25, 26]. Consequently, it is crucial to better investigate molecular drivers of metastasis to identify and develop new therapeutic targets for advanced‐stage BC. According to our findings, DARS2 affects BC cell growth, migration, invasion, apoptosis, and EMT through the PI3K/Akt/GSK‐3β/β‐catenin pathway.

DARS2 is increasingly reported with frequent overexpression within different human malignancies, like HCC [18], melanoma [27], and ovarian cancer [28]. Notably, previous research has demonstrated that elevated DARS2 levels are correlated with lymph node metastasis, tumor differentiation, TNM stage, and dismal prognostic outcome of LUAD [19, 29]. Additionally, it has been reported that silencing DARS2 significantly impairs esophageal cancer cell growth, apoptosis, migration, and cell cycle progression [21]. But DARS2's precise effect in BC remains largely unexplored. The present work combined bioinformatic analysis with clinical validation for investigating DARS2 expression and its clinical implications in BC. Our findings revealed that DARS2 expression dramatically increased within both BC cells and tissues. Importantly, we suggested that DARS2 overexpression was closely linked to advanced tumor stages, lymph node metastasis, molecular subtypes, and unfavorable overall survival of BC patients, suggesting that DARS2 may be the novel prognostic biomarker and the promising anti‐BC therapeutic target. Moreover, DARS2 knockdown effectively inhibited the aggressive behavior of BC cells, including proliferation, cell cycle progression, and promotion of apoptosis.

EMT is a critical process in cancer metastasis, which enables epithelial tumor cells to acquire mesenchymal properties, leading to enhanced motility and invasiveness [30]. From our results, DARS2 knockdown could upregulate E‐cadherin and downregulate N‐cadherin and vimentin to dramatically mitigate BC cell migration, invasion, and EMT, suggesting that DARS2 may facilitate BC metastasis by promoting EMT, thereby contributing to the aggressive behavior of BC cells. These findings are in accordance with prior results from additional cancers, such as bladder cancer [20] and LUAD [31], where reduced DARS2 expression is related to decreased cell invasion and migration. After DARS2 was knocked down, tumor growth and lung metastasis were suppressed in vivo, further underscoring that DARS2 inhibition may be the new anti‐BC treatment strategy. Given DARS2's critical role in multiple cancer types, therapies aimed at inhibiting DARS2 may have broad applicability in tumor treatment.

The PI3K/Akt pathway, as the well‐established oncogenic pathway, exerts an essential effect on modulating different cellular events, such as cell proliferation, survival, and metabolism [32, 33]. GSK‐3β and β‐catenin, the downstream components of this pathway, are associated with regulating tumor metastasis and EMT [34, 35]. Mechanistically, DARS2 exerted its oncogenic effects in BC cells through PI3K/Akt/GSK‐3β/β‐catenin pathway activation. DARS2 knockdown apparently suppressed PI3K and Akt phosphorylation, and downregulated GSK‐3β and β‐catenin. Consistent with our findings, DARS2 downregulation within LUAD cells is recently suggested to decrease PCNA and N‐cadherin levels, elevate cleaved‐caspase 3 and E‐cadherin levels, and inactivate the PI3K/AKT pathway [36]. Our rescue experiments using 740Y‐P further confirmed that adding 740Y‐P effectively abolished the impacts of DARS2 knockdown against BC cell growth, migration, invasion, apoptosis, as well as cell cycle progression. DARS2 knockdown in vivo was associated with decreased key protein levels related to the PI3K/Akt/GSK‐3β/β‐catenin pathway, confirming the mechanistic association between DARS2 and this pathway.

This work sheds valuable lights on DARS2's effect on BC, yet several limitations must be noted. First, the relatively small sample size of clinical specimens necessitates future validation with more samples for strengthening the clinical significance of our findings. Second, the exact mechanisms inducing DARS2 upregulation within BC are still unknown, warranting further investigation into potential contributors like dysregulated transcriptional networks, epigenetic modifications, or post‐transcriptional regulation. Moreover, dynamic changes in DARS2 subcellular localization (mitochondria vs. cytoplasm) during tumor progression are not explored, probably limiting a holistic understanding of its mechanistic involvement in PI3K/Akt pathway regulation. Lastly, direct binding of DARS2 to critical PI3K/Akt pathway components (e.g., PI3K regulatory subunits or Akt) has not been experimentally validated through methods such as co‐immunoprecipitation (Co‐IP), resulting in indirect mechanistic interpretations. These limitations highlight critical avenues for future studies to fully elucidate the multifaceted role of DARS2 in BC progression.

Collectively, DARS2 expression increases within BC, which exerts an important effect on enhancing tumor progression through the PI3K/Akt/GSK‐3β/β‐catenin pathway. From these observations, DARS2 is a candidate new prognostic biomarker and promising anti‐BC therapeutic target.

## Conflicts of Interest

The authors declare no conflicts of interest.

## Data Availability

The data that support the findings of this study are available from the corresponding author upon reasonable request.
